# Die Rolle der Chemotherapie im Kontext der Bestrahlung beim Nasopharynxkarzinom: Update der MAC-NPC-Netzwerk-Metaanalyse

**DOI:** 10.1007/s00066-023-02164-9

**Published:** 2023-10-19

**Authors:** Alexander Rühle, Nils H. Nicolay

**Affiliations:** 1 Klinik und Poliklinik für Strahlentherapie, Universitätsklinikum Leipzig, Stephanstr. 9a, 04103 Leipzig, Deutschland; 2 Arbeitsgruppe junge DEGRO der Deutschen Gesellschaft für Radioonkologie e. V. (DEGRO), Berlin, Deutschland; 3 Mitteldeutsches Krebszentrum (CCCG), Partnerstandort Leipzig, Leipzig, Deutschland

## Hintergrund und Ziel der Arbeit

Lokoregionär fortgeschrittene Nasopharynxkarzinome sollten mit einer simultanen Radiochemotherapie behandelt werden, wobei eine Intensivierung der Systemtherapie, entweder durch Induktionschemotherapie oder adjuvante Chemotherapie, möglicherweise die onkologischen Ergebnisse weiter verbessern kann. Während die vergangenen MAC-NPC-Metaanalysen die Therapiesequenz einer simultanen Radiochemotherapie gefolgt von einer adjuvanten Chemotherapie hinsichtlich des Gesamtüberlebens favorisierten, sind zuletzt neuere Studien veröffentlicht worden, welche potenziell größere Vorteile für die Sequenz einer Induktionschemotherapie gefolgt von einer simultanen Radiochemotherapie zeigten, sodass nun eine aktualisierte Metaanalyse der MAC-NPC-Gruppe auf Basis individueller Patientendaten erfolgte.

## Patienten und Methoden

Für diese Metaanalyse wurden randomisierte Studien zum nichtmetastasierten Nasopharynxkarzinom, welches mit Radiotherapie allein oder kombiniert mit (induktiver, simultaner oder adjuvanter) Chemotherapie behandelt wurde, in allgemeinen Datenbanken wie *PubMed* und *Web of Science* sowie chinesischen medizinischen Literaturdatenbanken identifiziert; für die Analyse wurden individuelle Patientendaten verwendet.

## Ergebnisse

Die aktualisierte Netzwerkmetaanalyse umfasste 28 Studien mit insgesamt 8214 Patienten; die berücksichtigten Studien rekrutierten zwischen 1988 und 2016. Die mediane Nachbeobachtungsdauer betrug 7,6 Jahre. Die drei Therapieformen mit dem höchsten Nutzen für das Gesamtüberleben waren eine taxanhaltige Induktionschemotherapie gefolgt von einer Radiochemotherapie (HR = 0,75; 95 %-KI 0,59–0,96), eine taxanfreie Induktionschemotherapie gefolgt von einer Radiochemotherapie (HR = 0,81; 95 %-KI 0,69–0,95) und eine Radiochemotherapie gefolgt von einer adjuvanten Chemotherapie (HR = 0,88; 95 %-KI 0,75–1,04), jeweils in Bezug zur alleinigen simultanen Radiochemotherapie. Die Wahrscheinlichkeit für eine Verbesserung des Gesamtüberlebens durch diese Therapien im Vergleich zu den übrigen Therapiesequenzen wurde mit dem sogenannten P‑Score beziffert: Dieser betrug 92 % für eine zusätzliche taxanhaltige Induktionschemotherapie, 87 % für eine taxanfreie Induktionschemotherapie und 72 % für eine adjuvante Chemotherapie.

## Schlussfolgerung der Autoren

Die Therapieintensivierung mittels Induktionschemotherapie oder adjuvanter Chemotherapie verbessert das Gesamtüberleben gegenüber einer alleinigen simultanen Radiochemotherapie bei Patienten mit fortgeschrittenem Nasopharynxkarzinom. Weitere randomisierte Studien sind jedoch notwendig, um im direkten Vergleich die Überlegenheit einer Induktionschemotherapie oder adjuvanten Chemotherapie weiter zu quantifizieren.

## Kommentar

Den Autoren der MAC-NPC-Gruppe muss zu dieser aktualisierten Metaanalyse mit 28 Studien und über 8000 Patienten gratuliert werden, vor allem aufgrund der umfangreichen statistischen Analysen inklusive Sensitivitätsanalysen, welche die Robustheit der Ergebnisse erhöhen. Die Fragestellung ist klinisch relevant und angesichts der Häufigkeit von Nasopharynxkarzinomen (ca. 130.000 Neuerkrankungen mit 80.000 Todesfällen pro Jahr [1]) von globalem Interesse.

Die simultane Radiochemotherapie bildet den aktuellen Therapiestandard beim Nasopharynxkarzinom in den UICC-Stadien III und IV und ist einer alleinigen Radiotherapie überlegen. Allerdings kann eine Intensivierung der systemischen Therapie, entweder mittels Induktionschemotherapie oder adjuvanter Chemotherapie, die onkologischen Ergebnisse beim lokoregionär fortgeschrittenen Nasopharynxkarzinom weiter verbessern [2]. Durch die Inklusion neuerer Studien in die MAC-NPC-Datenbank zeigten nun drei intensivierte Therapieschemata (taxanhaltige Induktionschemotherapie, taxanfreie Induktionschemotherapie, adjuvante Chemotherapie) einen Vorteil hinsichtlich des Gesamtüberlebens verglichen mit einer alleinigen simultanen Radiochemotherapie bei Patienten mit lokoregionär fortgeschrittenen Nasopharynxkarzinomen. Auch in Bezug auf die lokoregionäre Tumorkontrolle, das progressionsfreie Überleben und das krebsspezifische Überleben waren diese drei systemtherapeutisch intensivierten Schemata einer alleinigen simultanen Radiochemotherapie überlegen. Hinsichtlich der Fernmetastasierungsrate wiesen Therapiesequenzen mit einer (taxanfreien) Induktionschemotherapie die besten Ergebnisse auf; die Inzidenz von Fernmetastasen war geringer als bei Konzepten mit adjuvanter Chemotherapie. Es erfolgte auch eine präspezifizierte Analyse einer möglichen Altersabhängigkeit der Ergebnisse: Bezüglich des Gesamtüberlebens zeigte sich – anders als bei der MACH-NC-Metaanalyse zu Kopf-Hals-Plattenepithelkarzinomen anderer Lokalisationen [3] – keine Interaktion zwischen dem Alter und dem Therapieeffekt für die (in dieser Metaanalyse relevanten) Therapiesequenzen Induktionschemotherapie plus simultane Radiochemotherapie beziehungsweise Radiochemotherapie plus adjuvante Chemotherapie. An dieser Stelle muss aus unserer Sicht jedoch kritisch angemerkt werden, dass der geringe Anteil älterer Patienten (etwa 10 % über 60 Jahre) in der MAC-NPC-Datenbank die Extrapolation der Ergebnisse auf ältere Patienten mit Nasopharynxkarzinom, wie sie in europäischen Ländern häufiger vorkommen, deutlich einschränkt.

Verglichen mit Voranalysen der MAC-NPC-Datenbank, bei denen die simultane Radiochemotherapie gefolgt von einer adjuvanten Chemotherapie bei Patienten mit fortgeschrittenem Nasopharynxkarzinom den absolut größten Vorteil in Bezug auf das Gesamtüberleben aufgewiesen hatte [2], konnte nun eine mindestens onkologische Gleichwertigkeit der Induktionschemotherapie mit der adjuvanten Chemotherapie (jeweils kombiniert mit der simultanen Radiochemotherapie) festgestellt werden – im Trend weist sogar die Induktionschemotherapie den größeren Überlebensvorteil als die adjuvante Chemotherapie auf.

Signifikant beeinflusst wurden diese Ergebnisse durch die Berücksichtigung der NPC-0501-Studie, einer Studie mit kompliziertem und im Verlauf der Studie geändertem Randomisierungsalgorithmus, bei der neben der vergleichenden Analyse zwischen einer Induktionschemotherapie und einer adjuvanten Chemotherapie auch ein Vergleich zwischen normofraktionierter und moderat akzelerierter Radiochemotherapie erfolgt war [4]. In dieser Studie zeigte sich ein signifikant verbessertes progressionsfreies Überleben und Gesamtüberleben in der Induktionschemotherapiegruppe verglichen mit der adjuvanten Chemotherapiegruppe, was vor allem durch eine Reduktion der Fernmetastasierungsrate bedingt war. Zu bedenken ist bei der Frage Induktionschemotherapie versus adjuvante Chemotherapie, dass die Therapieadhärenz der adjuvanten Chemotherapie geringer ist, beispielsweise konnten in der Intergroup-0099-Studie nur 55 % der Patienten alle 3 Zyklen der adjuvanten Cisplatin/5-FU-Chemotherapie erhalten [5]. Insbesondere ältere Patienten mit Nasopharynxkarzinom können häufig keiner adjuvanten Chemotherapie mit 3 Zyklen Cisplatin/5-FU zugeführt werden. Die vielbeachtete und hochrangig in *The Lancet* publizierte Studie zum Stellenwert einer adjuvanten metronomischen Capecitabin-Erhaltungstherapie nach einer Radiochemotherapie schloss beispielsweise gar keine Patienten über 65 Jahre ein, sodass die Datengrundlage für ein solches Vorgehen in dieser vulnerablen Patientengruppe limitiert ist [6]. Die Tatsache, dass die üblicherweise verabreichten Induktionschemotherapien (Cisplatin/Gemcitabin, Cisplatin/5-FU/Docetaxel, Cisplatin/5-FU) Cisplatin beinhalten, ist ein weiterer Grund dafür, dass eine Induktionschemotherapie bei einer signifikanten Anzahl älterer Nasopharynxkarzinompatienten aufgrund der verglichen mit jüngeren Patienten höheren Prävalenz von Cisplatin-Kontraindikationen nicht durchgeführt werden kann.

Wichtig ist zu erwähnen, dass eine Induktionschemotherapie aufgrund des relativ hohen Risikos für höhergradige Toxizitäten wie beispielsweise Leukopenie und Neutropenie an Zentren mit suffizienter Erfahrung für solche Konzepte erfolgen sollte. Insbesondere eine Verzögerung oder gar Verhinderung der folgenden cisplatinbasierten Radiochemotherapie muss unbedingt vermieden werden, um die Heilungschancen nicht zu kompromittieren – die alleinige konkomitante Radiochemotherapie sorgt bereits für relativ hohe Heilungschancen (5-Jahres-Überleben von etwa 70 % [2]) und bleibt somit der entscheidende Therapiebaustein. Bei lokal sehr fortgeschrittenen Tumoren mit unmittelbarer Nähe des Tumors zu kritischen Risikoorganen wie den optischen Strukturen kann eine Induktionschemotherapie eine Tumorverkleinerung bewirken, sodass möglicherweise erst dadurch die Dosisgrenzwerte der Risikoorgane eingehalten werden können – wenn ein solches Vorgehen durchgeführt wird, sollte allerdings das in der Konturierungskonsensusarbeit von Lee et al. genannte Prozedere (u. a. Inklusion des initialen [prächemotherapeutischen] Tumors zumindest im intermediären Risiko-CTV mit 64 Gy) beachtet werden [7].

Für die tägliche klinische Praxis bleibt die wichtige Frage, inwieweit die Ergebnisse dieser Metaanalyse übertragbar sind auf europäische Länder, in denen Nasopharynxkarzinome nicht endemisch sind und auch andere histopathologische Eigenschaften aufweisen (häufiger Plattenepithelkarzinome statt EBV-assoziierte undifferenzierte Karzinome). Eine große europäische Kohortenanalyse konnte beispielsweise keinen Vorteil einer Induktionschemotherapie bei Patienten mit EBV-negativen Nasopharynxkarzinomen zeigen [8]. Dessen ungeachtet empfiehlt die aktuelle National-Comprehensive-Cancer-Network(NCCN)-Leitlinie für lokoregionär fortgeschrittene Nasopharynxkarzinome präferenziell eine Induktionschemotherapie gefolgt von einer Radiochemotherapie (Kategorie 1), alternativ wird aber auch eine alleinige Radiochemotherapie oder eine Radiochemotherapie gefolgt von einer adjuvanten Chemotherapie genannt (jeweils Kategorie 2B; [9]). Die europäischen Leitlinien machen divergente Empfehlungen bezüglich einer Induktionschemotherapie oder adjuvanten Chemotherapie bei lokoregionär fortgeschrittenen Nasopharynxkarzinomen: Während die britische Leitlinie [10] eine alleinige simultane Radiochemotherapie präferiert, nennen sowohl die aktualisierte EHNS-ESMO-ESTRO- [11] als auch die aktualisierte spanische Leitlinie [12] die Induktionschemotherapie und adjuvante Chemotherapie als Optionen. Zukünftige Anpassungen der Leitlinien unter Berücksichtigung der hier diskutierten Metaanalysedaten bleiben abzuwarten. *Head-to-head*-Vergleiche zwischen einer Induktionschemotherapie und einer adjuvanten Chemotherapie erfolgen aktuell in Form von randomisierten Studien (NCT03306121; NCT01797900).

Einige wenige Limitationen dieser Metaanalyse werden von den Autoren aufgeführt. Die Inklusion von Studien mit Studienbeginn im Jahr 1988 führt natürlich dazu, dass viele Patienten mit heute als veraltet anzusehenden Bestrahlungstechniken behandelt wurden. Eine möglicherweise prädiktive Rolle der EBV-DNA-Plasmakonzentration wurde in dieser Metaanalyse nicht untersucht, da diese Daten nicht für alle Studien verfügbar waren. Möglicherweise können die DNA-Konzentration von EBV im Plasma und die peritherapeutische Dynamik dieses Biomarkers unter der Therapie zukünftig zur Therapieindividualisierung der zusätzlichen systemtherapeutischen Bausteine beitragen, allerdings bedarf es hierzu weiterer Studien und standardisierter Methoden zur EBV-DNA-Plasmamessung.

Abschließend ist festzuhalten, dass basierend auf dieser qualitativ hochwertigen Metaanalyse eine Intensivierung der Chemotherapie als Induktion oder Adjuvans bei Patienten mit Nasopharynxkarzinom im Stadium III oder IV zumindest bei gutem Allgemeinzustand auch in nicht endemischen Regionen erfolgen sollte, insbesondere wenn die Tumoren undifferenzierte EBV-positive Karzinome sind und/oder eine große Tumorlast aufweisen. In der Zukunft ist die Etablierung eines risikoadaptierten Vorgehens basierend auf neuen Prognosemarkern (z. B. EBV-Titer, neue Risikofaktoren in der Bildgebung wie tiefsitzende zervikale Lymphknoten) wünschenswert, um gezielt Patienten zu identifizieren, welche von der Intensivierung der Systemtherapie profitieren, und umgekehrt diejenigen zu erkennen, denen die zusätzlichen Toxizitäten erspart werden können.

*Alexander Rühle, Nils H. Nicolay, Leipzig*
